# The extent to which family doctors have the ability to deal with patients with disabilities

**DOI:** 10.3389/fmed.2025.1524123

**Published:** 2025-04-16

**Authors:** Ohud Adnan Saffar

**Affiliations:** Department of Special Education, College of Education, King Saud University, Riyadh, Saudi Arabia

**Keywords:** intern family doctors, special education, the Kingdom of Saudi Arabia, patients with disabilities, questionnaire

## Abstract

Lack of training of health workers presents a unique challenge in meeting the needs of patients with disabilities (PWD). Therefore, the current study aimed to determine the level of medical students’ ability to deal with PWD in hospitals by knowing the IFD ‘attitudes and the challenges they face while working with this group. Additionally, the study sought to understand the intern family doctors’ (IFD) perspectives on problem-solving strategies. To achieve the study objectives, a questionnaire was designed to measure trends, challenges, and solutions to the problems facing IFD. The study sample comprised 152 doctors from various medical colleges in Riyadh. At this point, the data underwent descriptive analysis, and the ordinal alpha indicated a reliability of 0.71 for the internal consistency measure. The study found that IFD have a median ability to deal with PWD, with a median score of 2.37. IFD have positive attitudes, with a median score of 2.27. However, trainee IFD face difficulties in communication, proficiency, implementing rights and legislation, and attending special education training workshops (a median 2.21). The study found a positive correlation between solutions to these problems and the enhancement of medical students’ understanding of PWD characteristics and strategies, with a median score of 2.63. However, the multiplicity of areas in the Kingdom of Saudi Arabia (KSA) hinders the generalization of the results. Finally, participants recommended establishing special education diplomas and curricula for IFD’, ensuring the presence of a PWD specialist in clinics to address doctors’ challenges, and providing periodic training workshops for doctors to attend.

## Introduction

The provision of care for patients with disabilities (PWD) poses a significant challenge to healthcare professionals due to the multiple care demands. Where barriers include communication barriers, financial barriers, medical care barriers, high effort and time barriers, misconception barriers, and universal accessibility barriers (1). Also, medical professionals require additional time to treat children with PWD, especially where there are developmental disabilities that limit self-control of behavior and communication due to developmental delays and cognitive disabilities. Studies by Clemente et al. (2) and Barart and Taaka (3) confirm that PWD face multiple barriers in accessing health services. These barriers include communication issues; financial issues; psychological, behavioral, and attitudinal issues; scarcity of service provision; and organizational and transportation barriers. Misuse of financial resources, lack of equipment, and insufficient physical and human resources are the main obstacles to coverage and universal access to health services. Hamilton et al. (4) reported that the challenges faced by PWD included arranging visits with both primary care physicians and specialists due to limited availability, long waiting periods even for urgent cases, insurance barriers, transportation issues, and difficulties in obtaining basic pain relievers. The study also noted that these issues are more prevalent in rural areas, with financial problems and a lack of health insurance being the most prominent. Lezzoni et al. (5) also demonstrated that uninsured PWD face significantly greater barriers to accessing health care than insured PWD. Others also have mobility, attention, and cooperation problems and hence require special attention (6, 7). According to Sharby et al. (8) and Doebrich et al. (9), healthcare professionals must adopt alternative communication methods with children with PWD in their facilities. The inability of physicians to understand sign language and Braille and the lack of knowledge about the impact of the use of assistive devices such as hearing aids significantly impact the caregiver’s ability to deal with and manage the care of people with disabilities (10, 11). Without the proper education and skills preparation, it is challenging for caregivers to adjust and accommodate the unique needs of the PWD while ensuring optimal care and the best possible outcomes (10). Therefore, there is a need for additional and enhanced professional development and empowerment to ensure that doctors and other caregivers within the hospital setting can interact with and care for clients who are PWD (12). Studies underscore the widespread negative attitudes towards treating individuals with disabilities in clinical settings, often stemming from societal pressures and beliefs. These attitudes can lead to stigma, neglect, and misconceptions, resulting in unfavorable patient care environments (13, 77). The practice of integrating disability awareness workshops in hospitals, as well as a disability-focused curriculum of education, can help mitigate these problems (14). Further, a proactive approach focused on professional development and the empowerment of the intern family doctors (IFD) will help them acquire the necessary skills and positive attitude to deal with the complexities of managing PWD (12). One proposed solution to this problem is integrating the study of special education as part of the curriculum in medical colleges and institutions (12). Such programs can help equip experts with the skills to recognize and approach disability in their clients from an informed and empowered position and deal with them most respectfully (9). United States of America (USA) and the United Kingdom (UK) are two notable countries that have adopted the curriculum approach, and the integration not only confers skills and expertise but also improves the attitudes of the students toward PWD and helps them deal with other forms of diversity in the clinical setting (15). Medical schools in the USA, UK, and other countries are enhancing PWD care by incorporating simulated interactions, interdisciplinary teamwork, and community-based learning (16, 78). An example of a methodology for developing health student professionals to deal with PWD is in the United States. Clarke et al. (17) developed 15 elective courses on disability, incorporating the perspectives of PWD into a mandatory curriculum for doctor training. There was also talk about a disability-focused medical curriculum in the United States in 2021, according to Pathmathasan (18). This curriculum would teach medical students about the ethics of care, positive medical and social models of disability, and intersectionality. As medical students learn these skills, they will help create a medical community that sees their patients as people. Despite its success ratings and the educational program’s accolades from countries that have been successful, countries like the Kingdom of Saudi Arabia (KSA) have yet to adopt this integration. Due to this reluctance, medical professionals from their institutions lack the necessary skills, knowledge, and attitude to effectively care for clients with PWD (7). There is a need for a broader scope of implementation of the special-needs curriculum in medical schools to bridge the existing knowledge and practice gap.

Therefore, this study aims to reveal IFD attitudes toward the treatment of PWD. Additionally, comprehending the challenges IFD encounter when assisting PWD is crucial. Moreover, this study aims to propose suitable solutions for the problems faced by IFD who work with PWD. This information will determine the extent to which IFD or other doctors are able to treat and deal with PWD.

### Study questions

1. What is the level of the intern family doctors ‘s attitudes toward treating patients with disabilities?
2. What is the level of the challenges that the intern family doctors face when working with patients with disabilities?
3. What are the appropriate solutions to the problems that the intern family doctors encounters when working with patients with disabilities?

#### Importance of the study

1. The integration of the study of special education with health disciplines is being pursued to enhance the quality of life for PWD, thereby keeping pace with modern research and promoting exchange of experiences.
2. There is a dearth of research in KSA and the Arab world that explores the health sector’s capacity to assist PWD and deliver suitable services to them.
3. The health sector needs to receive assistance in enhancing the academic skills of its cadres and their capacity to interact with individuals who are PWD in a behavioral, psychological, and social context.
4. A new curriculum needs to be created to educate IFD in the Faculty of Medicine and other specializations on how to deal with PWD in terms of psychological, linguistic, communication, and health.

### Study terms

#### Intern family doctor

They are medical students who have completed their bachelor’s degree and in the field of family medicine work alongside physicians in clinical settings during their third or fourth year. After graduation, they undergo additional years of internship and residency training before practicing independently. The internship year begins the first year of post-medical school training, and interns can practice medicine under supervision (19).

#### Patients with disabilities

Disability arises from the interplay between individuals with health conditions, such as cerebral palsy, Down syndrome, and depression, and personal and environmental issues, including adverse attitudes, inadequate transportation and public facilities, and insufficient social support. Furthermore, an individual’s environment significantly influences the experience and degree of disability. Inaccessible surroundings establish obstacles that frequently impede the complete and effective involvement of individuals with disabilities in society on an equal footing with others. Advancements in enhancing social participation can be achieved by mitigating these obstacles and supporting individuals with impairments in their daily activities (20).

## Literature review

### Laws and regulations in place to care for patients with disabilities

The Convention on the Rights of PWD (CRPD) is a comprehensive international legal instrument that emphasizes equality, accessibility, and the recognition of disability rights as fundamental human rights (21). It includes key provisions related to health care, such as equal access, disability-specific services, inclusive sexual and reproductive health, informed consent, and rehabilitation (22). The European Accessibility Act (2019) mandates accessibility in healthcare facilities and services, while the European Disability Strategy 2021–2030 aims for equal access to healthcare for PWD (22). Moreover, national and regional initiatives, such as the Disability Discrimination Act (DDA) in the UK, the Americans with Disabilities Act (ADA) in the U.S., and the Disability Act in Ireland, also play vital roles in shaping disability-inclusive health care systems worldwide (21, 23).

KSA has implemented several laws and initiatives to support PWD, ensuring equal access to healthcare, education, rehabilitation, and employment opportunities. Also, the importance of providing assistive devices and facilitating medical care, mandating the provision of healthcare services to PWD (24). The General Authority for Disability Affairs oversees the implementation of disability rights and laws, focusing on improving the quality of life for PWD citizens (25). The APD (26) and KSA’s Ministry of Education are working towards a vision of improved healthcare services for the visually impaired. The Social Security System provides financial assistance and health insurance programs for PWD, while KSA has a network of rehabilitation centers. The National Transformation Program (Vision 2030) aims to modernize healthcare systems, improve accessibility, and incorporate disability services. The Ministry of Education collaborates with healthcare services to ensure PWD access to medical care within the educational system (25, 26).

### Challenges facing intern family doctor when dealing with patients with disabilities

PWD, as described by Keller (7), face unique challenges in medical practice, including effective communication obstacles, as PWD may struggle with non-verbal communication, cognitive impairments, and auditory or visual impairments. Bu et al. (27) highlight the need for appropriate accommodations, such as interpreters, written materials, or assistive technology, to improve patient experience and care quality. Moreover, healthcare facilities often lack adequate facilities for PWD, such as wheelchair-accessible examination rooms and adaptive equipment, and transportation challenges can make it difficult for them to access medical appointments (7, 28). In addition, the complex health conditions of PWD represent one of the barriers facing IFD; they often require specialized care and multidisciplinary collaboration from IFD (12). Also, healthcare providers may unconsciously harbor biases and misconceptions toward PWD, leading to inaccurate assessments of their quality of life and capacity to participate in care. Diagnostic overshadowing can occur when doctors attribute all symptoms to the disability (12, 28).PWD often faces challenges in care coordination due to the need for multiple specialists and bureaucratic hurdles in the healthcare system (28). Additionally, doctors often lack adequate disability training, which focuses on understanding patient needs, communication techniques, and adaptive tools. This lack of training can also hinder access to necessary treatments, therapies, or medications for PWD (7, 9, 12). Also, Lee et al. (79) highlight the time constraints faced by healthcare providers when dealing with PWD, highlighting the need for longer consultations, adjustments in communication techniques, and the need for additional coordination efforts and assistive technology. Furthermore, PWD faces financial and insurance barriers, including uninsured services like physical therapy and home care. Long-term or specialized care can be financially burdensome, making it difficult for doctors and patients to navigate (9). Improving healthcare accessibility for PWD requires professional training, access to assistive technologies, and systemic reforms (7, 12).

### Intern family doctor’s attitudes toward patients with disabilities

Research has shown that medical professionals, including IFD, often hold negative attitudes toward PWD, which can affect the quality of care provided. The findings of several key studies underscore this issue. Medical professionals frequently lack confidence in providing equal care to PWD, which can negatively impact the quality of care they provide (10, 14). Limited exposure and education on disability issues in IFD exacerbate this lack of confidence (29). Often doctors and interns have limited experience and understanding of the specific needs of PWD. Additionally, medical professionals frequently exhibit implicit biases and stereotypes, which can result in incorrect treatment options (6, 30). Therefore, Long-Bellil et al. (28) advocate for disability-inclusive training, direct patient interaction, and addressing systemic biases in healthcare education to guarantee equitable healthcare for PWD and to enhance attitudes.

A study by Iezzoni et al. (31) revealed that merely 40.7% of doctors felt at ease delivering quality care to those with PWD, while only 56.6% exhibited a welcoming attitude toward them. The IFD identify the absence of accessible facilities and equipment and provider bias as an obstacle to providing care for PWD, particularly those with mobility impairments (32, 33). Moreover, disability experts, who have committed their efforts to working with PWD or on disability issues, exhibit both overt and covert biases favoring non PWD (34). The findings regarding bias and discomfort toward PWD underscore the necessity for comprehensive disability awareness education throughout the medical education continuum.

To narrow down our search, in the KSA, doctors’ attitudes toward patients with PWD vary, despite numerous studies highlighting persistent challenges in providing equal care. Al-Abdulwahab and Al-gain (35), demonstrate that numerous Saudi healthcare professionals, akin to their international peers, frequently adhere to a medical model of disability, prioritizing treatment and rehabilitation above the social model that promotes integration and equality. This may result in an insufficient comprehension of the social obstacles encountered by PWD, thereby affecting the delivery of care. Also, Woodman et al. (33) indicated that healthcare providers exhibit slightly negative attitudes toward people with disabilities, fostering biases in the delivery of care.

In contrast, there are a number of initiatives to improve IFD’ attitudes towards PWD and to improve health care. Where Sinha et al. (29) confirmed the need to teach a comprehensive disability awareness curriculum to second-year IFD. This curriculum should include disability etiquette, patient-centered, and interprofessional learning sessions for PWD as well as intellectual and developmental disabilities. Additionally, the results indicate that the curriculum improved the attitudes of IFD toward PWD after their post-curriculum training. Thus, the curriculum equips future doctors to address the unique needs of PWD. It fulfills essential competencies, offers opportunities for learning in interprofessional settings, and incorporates PWD into the educational framework.

Moreover, the US Department of Education (36) reported that, despite having completed two-thirds of a curriculum rich in disability-related content, IFD had enhanced attitudes toward wheelchair users. This improvement occurred after the department of physical therapy students, acting as interprofessional peer educators, instructed IFD on wheelchair mobility. Shakespeare et al. (37) confirmed the effectiveness of the “Come Roll with Me” (CRWM) initiative in implementing established educational programs that foster awareness and empathy for PWD, including the use of wheelchair users as facilitators. This study wanted to improve CRWM by creating, implementing, and testing a mixed-methods disability awareness curriculum within a UME preclinical course. The goal was to change the way IFD felt about people with disabilities (38, 39).

### Challenges facing patients with disabilities in the medical field

PWD faces numerous barriers to adequate healthcare, including physical barriers such as reduced access to services, lower rates of preventive care screenings, and worse health outcomes compared to the general population (40). The knowledge and attitudes of healthcare professionals also contribute to these barriers, as PWD often have a distrust and skepticism towards medical professionals (41). Additionally, Guidry-Grimes et al. (42) also observed that PWD faces barriers due to the knowledge and attitudes of healthcare professionals, as well as distrust and skepticism towards medical professionals. Also, PWD also face significant issues related to current healthcare management, with healthcare professionals often assuming PWD have a lower quality of life, but their self-reported experiences often contradict this (1, 37). Moreover, recent research, including the study by Asiri et al. (43), suggests that the diminished quality of life for PWD is frequently attributable not to the disability itself, but to societal and environmental obstacles, including insufficient accessibility, discrimination, and inadequate healthcare services. Also, They PWD often face communication difficulties with healthcare providers, which can be particularly difficult for patients with communication difficulties. Physical barriers related to access and transportation can also prevent PWD from attending necessary appointments (8). Therefore, it is crucial to educate medical practitioners, staff, and trainees about PWD’s essential characteristics and advocate for their children’s needs in pediatrics (9, 44).

In KSA, PWD face numerous obstacles when seeking medical and healthcare services. The initial obstacle is negative perceptions: Numerous healthcare professionals regard disability solely from a medical standpoint, emphasizing treatment above the wider social inclusion of persons. This viewpoint may result in biases in care provision, including diminished expectations on the quality of life for PWD, thereby influencing treatment options and decisions (26). The second obstacle is an absence of communication abilities. Healthcare personnel frequently lack the necessary training to engage effectively and empathetically with PWD. This may lead to emotions of dissatisfaction or marginalization among PWD (33). The third hurdle is the restricted access to expert care: there is a deficiency of specialized rehabilitation clinics, especially in rural regions. Urban locations house the majority of sophisticated healthcare facilities for PWD, thereby restricting access for those in more isolated areas (26). The fourth barrier comprises cultural and social obstacles: cultural attitudes in KSA can marginalize those with impairments, resulting in their exclusion from mainstream society. Families may conceal PWD from societal engagement, limiting their access to healthcare and support services (25).

### Educational training of IFD to deal with patients with disabilities

Undergraduate medical education (UME) provides an initial opportunity to cultivate the competencies and dispositions necessary for IFD to competently serve this demographic. According to Seidel and Crowe (45) and Holder et al. (46) the percentage of American medical schools that incorporate a disability awareness curriculum may be as low as 23%, which leaves IFD feeling unprepared to provide care for PWD. Additionally, the Association of American Medical Colleges (AAMC) records that medical schools possess the autonomy to determine how to integrate cultural competency and healthcare equity in the treatment of PWD into their curricula (47). While some schools use standardized patients to teach students how to conduct physical exams and interact with PWD, others have implemented structured clinical examinations with this population during the clerkship years to provide students with real-life experience (31, 48).

In contrast, the Parish et al. (49) study has proven that some medical schools currently lack a significant disability curriculum and a comprehensive approach to care for PWD. This result is similar to the study of Woodman et al. (33), which pointed out that many IFD in KSA lack adequate training regarding the specific needs of PWD. Also, research indicates that increased exposure to disability issues and targeted training may enhance these attitudes, as evidenced by educational interventions.

Furthermore, in KSA, there is an educational and training gap for healthcare workers. Many medical professionals do not receive adequate training to address the specific needs of PWD. Education about comprehensive care for PWD is often absent from medical school curricula, resulting in insufficient support for these patients (33). While there has been progress in many areas of medical education reform in KSA, the inclusion of special education and disability-specific curricula remains limited. A more comprehensive approach is necessary to prepare future IFD to provide inclusive and effective care for PWD (50).

Section 5307 of the Patient Protection and Affordable Care Act supports the results of previous studies by mandating curricula that educate health care workers or clinicians on how to deal with PWD. Additionally, in 2019, the Alliance for Disability in Health Care Education developed a set of core competencies on disability for health care education, which are mandatory for all practitioners (23). However, medical colleges do not impose such curriculum on their students. Subsequently, the neglected disability training of providers is a recognized shortcoming that exacerbates health care inequities (29).

## Methodology

### Research design

The study explores the views of IFD on treating PWD and the challenges they face in providing assistance. It aims to identify suitable solutions using a questionnaire to gather their views and suggestions, ensuring effective treatment and support for PWD. To achieve the objective, a descriptive design was used using a questionnaire. The study employs a questionnaire survey, suitable for obtaining explicit information from respondents who possess the knowledge and expertise to address questions pertaining to the phenomenon (51, 52). Self-reports collected through questionnaires frequently assess individuals’ views, attitudes, beliefs, and motives (51, 53). The creation of questionnaires is a crucial and intricate task (54). Formulating inquiries to obtain valid and reliable facts for theoretical evaluation and informed decision-making necessitates proficient research skills (55, 56).

### Questionnaire design and structure

The researcher designed the questionnaire, which included affirmative, negative, and balanced statements. We divided the tool into three parts. The questionnaire consisted of 3 items, 31 closed-ended sub-items, and one open question, all of which were designed to understand the attitudes of IFD towards PWD. This questionnaire also helps to reveal the challenges faced by these doctors when working with PWD and find solutions to these obstacles (see Appendix 2). The questionnaire asked participants to rate their responses on a 3-point scale, ranging from strongly yes (3) to strongly no (1). Participants respond to the questionnaire by selecting one of three options: yes, no, or somewhat, depending on how much the answer applies to them. Also, a 3-point Likert scale was used for data collection because it is appropriate for the nature of the research, providing clear answers and reducing hesitation or neutrality in responses. It also makes it easier for participants to make clear decisions, especially in studies that require simplified statistical analysis or deal with an audience that may have difficulty distinguishing between more detailed options (57, 58). Thus, the scale adequately captures the participants’ attitudes and challenges.

Section 1: Attitudes of IFD toward PWD: It consists of 17 short questions, including yes/no questions. The researcher quoted these items from the [Chadd and Pangilinan (59) as stated in Bu et al. (27)] scale and then adopted them. Additionally, the first study by Chadd and Pangilinan (59) used a five-point Likert scale. However, the current study only changed the wording and structure of the items from 1–17, not the whole scale (see Appendix 1). This is why the scale was made as a three-point Likert (yes/somewhat/no) to be simplify responses, reduce the potential for neutral answers, and accommodate the study population’s specific needs,’ etc. (57).

Section 2: The difficulties faced by IFD when working with PWD: the second item consists of 10 questions, including yes/somewhat/no questions.

Section 3: Proposed solutions to the challenges: it consists of 4 questions, including yes/somewhat/no questions.

To verify the validity of the questionnaire and in accordance with the methodologies followed in survey studies to ensure honesty and reliability. Face validity was used to ensure the clarity of the questions and their suitability to the study objectives, as face validity is characterized by ease of application, improving the clarity of the questions, and enhancing the credibility of the research tool (60). Therefore, the questionnaire was presented to four experts specialized in the field of special education to verify the validity of the tool and to ensure the suitability of the measured structures according to the research objectives. To reduce bias in the subjective data collected through the questionnaire, the questions were designed in a clear and direct manner to minimize the possibility of misunderstandings or inaccurate answers. We placed emphasis on ensuring the confidentiality of information. Additionally, the use of scales from previous studies enhances the reliability of the data (57). They made some linguistic and structural modifications, and then it was presented again to ensure the suitability of the content to the study topic. As for measuring reliability with the internal consistency measure, ordinal alpha relies on polychoric correlations to ensure the internal consistency of a 3-point Likert scale for the 17 attitude-related ordinals; an internal consistency coefficient of 0.71 indicates acceptable reliability for research purposes, suggesting that the items are reasonably correlated and the tool is suitable for measuring the intended constructs.

Research indicates that ordinal alpha, which is based on polychoric correlation, is more appropriate for ordinal data because it takes into account the nonlinear nature of variables and provides a more accurate estimate of internal consistency. Consequently, the use of ordinal alpha in this study enhances the accuracy and reliability of internal consistency analysis results, making it more consistent with the characteristics of the data used (61). Cronbach’s Alpha is one of the most common methods for measuring internal reliability, but it relies on Pearson correlations, which assume that data are normally distributed and on a continuous scale (62). However, for measures based on ordinal data, such as a three-point Likert scale, this assumption is not entirely true, which can lead to an inaccurate estimate of internal consistency (63).

### Sampling

All IFD in the internship stage, specializing in family medicine at Riyadh’s medical colleges (Saud University, Noura University, Imam University, Colleges of Medical Care, King Saud bin Abdulaziz University for Health Sciences, Riyadh Elm University, Al-Ghad International College for Health Sciences for female students, Medicine at Al-Faisal University, Al-Farabi Private College, among others), represent the study population. We chose family medicine due to its frequent supervision of all family members, handling of all cases, initial diagnosis of the patient’s illness, and subsequent referral to a specialist doctor. We also selected the city of Riyadh, because it is the capital of the Kingdom of KSA, as a representative sample for the study.

Sample selection: The sample was selected after contacting the Research Ethics Committee at King Saud University. After obtaining approval, the Ethics Committee contacted all colleges of medical care in Riyadh to facilitate the researcher’s task. Then, coordination took place between the researcher and officials in the relevant authorities to contact resident physicians via their private email. Letters of approval were sent explaining the importance and purpose of the study and how to answer the questionnaire, with a focus on maintaining the confidentiality of the information provided and not revealing their private data. After distributing the approvals to all family physicians in all relevant colleges of medical care, the sample was formed based on their approval and response to the attached questionnaire.

According to Calton and Hall (64) and Campbell et al. (65), sampling facilitates the study of general population characteristics; also, they advocate the use of purposive sampling. The total number of IFDs in all colleges is 232 doctors in Riyadh; thus, we selected 152 IFDs from various medical colleges (see Table 1) to explain the demographic, The doctors’ approval and response to the questionnaire determined the sample size. The participants received an email reminder and encouragement to participate three times every 2 weeks. There were a number of IFDs who did not cooperate and did not agree to answer the study questionnaire; the number of IFDs who did not respond was 80. Therefore, the current study sample is considered representative of the community, as it is more than 50% of the study community. We distributed the data in the first semester of 2024.

**Table 1:** Demographic data of the participants.

Gender and Number of participants	Grade	Experience With PWD	Caring for PWD	Training to deal with PWD
Male = 52	Excellent = 78	No = 104	No = 56	No = 126
Female = 100	Very good = 54	Yes = 48	Yes = 96	Yes = 24
	Good = 16			
	Acceptable = 4			

### Analysis of data

The statistical evaluation employed descriptive analysis. Results were synthesized, and tables were used to facilitate the presentation. The 2024 version (25) of SPSS Statistics was utilized for data analysis (66). The scale is a three-point Likert scale with a non-normal distribution; the data remains ordinal. Whereas Harpe (67) says that non-parametric methods may work better, especially when the response scale has fewer than five categories, even though Likert-type data are usually thought of as continuous. For individual rating items with numerical response formats comprising four or fewer categories, nonparametric methods are suitable. So, this study used nonparametric methods like Spearman’s rho, median, and interquartile ranges to make sure the methods were sound and make it easier to compare groups (58). According to recommended statistical practices, the median and interquartile range are preferred for non-normally distributed data and nonparametric tests, because they are less affected by outliers and skewness in the distribution (68).

## Results

This section of the study presents the results gathered from the recruited respondents. All results were produced by statistical analysis, employing various tests as outlined in the preceding section.

### First

The results of the study were analyzed based on the answers to the study questions, where the first question of the study focused on knowing attitudes of IFD towards PWD such as those with autism, Down syndrome, multiple disabilities, the deaf or hearing disabled, and the blind, etc.

Table 2 reveals, the study revealed that despite challenges such as obtaining a medical history and resource consumption, the majority of participants believe that PWD can improve with treatment (59%). They also believe that dealing with them properly can make healthcare more efficient (67%). However, 86% of participants stressed the need for additional training for healthcare providers about PWD. They also believed that society has a responsibility to care for them. Despite these challenges, only 11% of participants expressed negative attitudes towards PWD, believing that treating them is hopeless and that they do not contribute to society. The majority of participants believed that dealing with PWD properly can make healthcare more efficient (67%). Therefore, increased training may improve the experience of dealing with PWD.

**Table 2:** Attitudes of Intern family Doctors towards PWD.

N	Item	Percent %			Average	Frequency
		Yes	Some time	No		
1	I am happy to be with PWD.	51	36	11	2.4	3
2	If I had the choice, I would prefer to work with healthy patients instead of PWD	40	32	26	2.1	2
3	The community has the responsibility to provide care for PWD.	81	11	0	2.7	3
4	PWD improve with treatment	59	38	0	2.6	3
5	Medical care for PWD consumes a lot of resources	50	28	21	2.1	2
6	Obtaining a medical history from a PWD is often a disaster and very difficult.	13	52	34	1.8	2
7	PWD generally do not contribute much to society.	0	35	59	1.5	1
8	The healthcare costs for PWD are expensive compared to an average person, and this is unfair.	35	21	43	1.9	2
9	I will welcome PWD in my clinic.	92	7	1	2.9	3
10	If the PWD is handled properly, they can be seen quickly like any other patient.	67	26	7	2.6	3
11	The extent to which my PWD understand is valuable to me as a doctor.	96	4	0	2.9	3
12	PWD are better off in specialized care facilities.	28	43	27	2.9	2
13	The healthcare program provides appropriate compensation for the care of PWD	31	52	15	2.1	2
14	Treating PWD takes an extremely long time.	22	60	17	2.0	2
15	There is a need for more training to prepare healthcare practitioners to provide care for PWD	86	13	0	2.9	3
16	Caring for PWD is not enjoyable.	11	28	59	1.5	1
17	Treatment for PWD is hopeless.	0	11	82	1.1	1
	Total axis score	152 respondents				

In summary, the results indicate that despite some challenges such as difficulty obtaining medical history and increased resource consumption, the majority of participants believe that PWD can improve with treatment (59%) and believe that dealing with them properly can make healthcare more efficient (67%). The need for additional training (86%); this is consistent with the challenges faced by healthcare providers, suggesting that increased training may improve the experience of dealing with PWD.

### Second

The study highlights the challenges faced by doctors when collaborating with PWD, with 92–31% of participants stating a lack of ability and skill in communication, including sign language and Braille. They also perceive a lack of proficiency and a failure to allocate sufficient time during sessions for patients with PWD. 55% of participants believe that healthcare programs do not provide adequate services and effectively implement rights and legislation for PWD. 31% believe that the health system implements PWD rights well, but 47% are not aware of modern laws related to PWD within Vision 2030. 47% feel they are shortchanged by PWD, but 73% deny that they do not give PWD enough time during sessions. One of the most important obstacles affecting doctors’ treatment of PWD is the lack of attendance at special education training workshops. See Table 3.

**Table 3:** The difficulties faced by doctors when working with PWD.

N	Item	Percent			Average	Frequency
		Yes	Some time	no		
1	I feel I am falling short towards PWD.	47	34	18	2.3	2
2	I often do not give the PWD enough time in the session.	6	21	73	1.3	1
3	They do not have the ability and skill to communicate with PWD in general.	18	61	19	2.0	2
4	The healthcare program provides adequate services for all PWD.	30	50	19	2.1	2
5	Master sign language and Braille.	1	7	92	1.1	1
6	I know the behavioral, psychological, and social characteristics of PWD.	11	35	52	1.6	1
7	The health program implements the rights and legislation for PWD.	31	55	13	2.2	2
8	The doctor is knowledgeable about all the modern laws and regulations enacted by the state based on Vision 2030.	18	34	47	1.7	2
9	The doctor trains on how to deal with PWD.	14	35	50	1.6	1
10	I attended many training workshops in the field of special education.	5	9	85	1.2	1
	Total axis score	152 respondents				

In summary, the findings show that there is a two-way connection between being able to care for people with disabilities (PWD) and the need to develop better programs. These programs should help participants learn how to communicate with PWD, understand their social needs, and know the relevant rules and laws.

### Third

The IFD has identified solutions to improve the abilities of doctors dealing with PWD. 80–73% of respondents believe in establishing and preparing special education curricula for IFD and health sector workers. 80% support adding special education courses in medical schools and health services. Most physicians see integrating PWD education into their academic curricula as a radical solution. A special education specialist in every clinic would be beneficial in addressing challenges faced by doctors. However, 73% of participants do not attend periodic training workshops for doctors, indicating a need for specialized knowledge in special education for improving healthcare for PWDs (Table 4).

**Table 4:** The solutions to solve the problems which faced intern family doctors with PWD.

N	Item	Percent			Average	Frequency
		Yes	Some time	No		
1	The presence of a special education specialist in every session solves the problem that doctors are facing.	54	38	8	2.5	3
2	Attending training workshops for doctors periodically	2	25	73	2.7	3
3	Preparing diplomas in special education to teach the medical specialty	73	15	10	2.6	3
4	Establishing curricula in the field of special education for teaching medical students and health sectors.	80	17	3	2.8	3
	Total axis score	152 respondents				

The study acknowledges the challenges faced by persons with disabilities (PWD) and advocates for sustainable solutions through education and training, suggesting recommendations to enhance healthcare quality and physician competence in handling PWD.

### Descriptive statistics and correlation coefficients

Kaur et al. (69) define descriptive statistics as the summary of the characteristics of the gathered data. Table 5 illustrates the results of the descriptive statistics for the gathered responses. The median value for IFD trends is 2.27, perceived challenges are 2.21, and perceived solutions are 2.63. The Interquartile ranges for IFD trends are 0.21, while it is 0.34 for challenges and 0.38 for solutions to problems.

**Table 5:** Descriptive statistics.

Item	Median	Interquartile ranges	N
IFD trends	2.27	0.21	152
Challenges	2.21	0.34	152
Solutions	2.63	0.38	152
Over all median	2.37	0.19	152

The Spearman correlation was used to analyze relationships because the data distribution is not normal (70), where the correlation coefficients of all variables are−0.267 for IFD trend variables, which is a significant relationship >0.01, this value shows a weakly negative relationship between IFD attitudes and increased awareness of how to deal with PWD. However, it may also mean that the less negative IFD attitudes are toward PWD, the better they are at dealing with them. Also, the correlation coefficient for the variable of opinions on challenges is 0.287, so it is a strong significant value, which means that the more challenges faced by the IFD, the more problems there are in the inability to provide services to the PWD. Finally, the correlation coefficient for variables for suggesting solutions for the problem is 0.033, this indicates that the association is very weak, as this value indicates that there is no statistically significant association, as Table 6 shows the Spearman correlation. However, the Spearman correlation value of 0.833 indicates that the sample of IFD has a high ability to deal with PWD, as long as the degree of stability is greater than 0.70 according to (71).

**Table 6:** Spearman correlation.

Item	Spearman correlation	*N*
IFD trends	−0.267^**^	152
Challenges	0.287^**^	152
Solutions	0.033^**^	152
Over all	0.833	152

** Correlation is significant at the 0.01 level (@-tailed).

The Spearman correlation coefficient shows a strong positive association between the studied variables, indicating that more proposed solutions and positive attitudes towards PWD among IFD improve their ability to deal effectively with them. The fewer challenges faced by IFD when treating PWD in healthcare also enhance their ability to deal with them. The practical implications of this result suggest that enhancing knowledge and training can directly lead to improving the care provided to PWD. Developing specialized training programs may significantly impact the efficiency of IFD in dealing with PWD.

## Discussion

The study investigates the competence of IFD in treating and managing PWD, revealing their attitudes towards the treatment process. It emphasizes the challenges doctors face while assisting PWD and proposes solutions to address these issues, thereby enhancing their understanding and support. The study found that IFDs have a median ability to deal with PWD of 2.27, indicating that positive attitudes and fewer obstacles make them more capable of working with this group. A positive work environment can enhance their capacity to interact with PWD and contribute to the success of the health field. The majority of participants agreed that IFDs should acknowledge the importance of patients with PWD and agree that supplementary training is essential for preparing healthcare practitioners to provide care. However, most participants disagreed with the notion that PWD do not contribute to society, and the majority believed that PWD treatment is important.

This result can be attributed to religious teachings on care and inclusiveness being central to KSA culture, as Islam places at the forefront of its priorities the care of PWD and the protection of their rights (72, 73). The principles of Islam encourage mercy, justice, and inclusiveness, which include PWD. Furthermore, the National Transformation Program (Vision 2030) supports this outcome, aiming to improve healthcare services, especially for PWD, by modernizing healthcare systems, enhancing accessibility, and integrating disability services. The Ministry of Education partners with healthcare providers to guarantee that PWD have access to medical treatment inside the educational system (25, 26). In contrast, the APD (26) revealed that PWD in KSA face challenges in accessing medical and healthcare services due to negative perceptions of doctors, leading to biases in care provision and reduced expectations of quality of life, affecting treatment options and decisions.

On the other hand, PWD individuals often find working with non-PWD individuals unenjoyable and believe that treating them is not their responsibility. This is due to a lack of awareness, education, and training among IFDs in health colleges about PWD rights and laws. Effective interaction with PWD individuals is crucial, as understanding their characteristics is essential for effective communication and addressing the challenges faced by IFDs. The research by Iezzoni et al. (31) concluded that the majority of IFD do not feel comfortable providing quality treatment to those with disabilities. Furthermore, medical professionals frequently exhibit implicit biases and stereotypes, which can result in incorrect treatment options (6, 29, 30). Studies by Lagu et al. (14) and Al-Zboon and Hatmal (10), have demonstrated that IFD frequently lacks confidence in delivering equitable care to PWD, thereby impacting the quality of care rendered. Friedman’s research (34) has agreed that PWD, or Person with Disabilities, often face biases and discomfort in their medical education, necessitating comprehensive disability awareness education. This education addresses both overt and covert biases, as many medical professionals lack experience and understanding of PWD’s unique needs. Sinha et al. (29) found a solution to these biases by creating an awareness program to educate health cadres on how to deal with PWD. The results of this study show that the awareness-raising approach helps provide effective disability education for medical students to prepare future physicians to serve PWD and their unique needs. In addition, both studies by McMillan et al. (32) and Iezzoni et al. (31) have confirmed that doctors recognize the lack of accessible facilities and equipment as a barrier to providing treatment for PWD, especially those with mobility impairments.

The challenges doctors encounter when dealing with PWD are many. The results showed that there are multiple difficulties that trainee doctors suffer from-on a median 2.21. The results also showed that these challenges affect doctors’ ability to work with PWD negatively. Furthermore, the majority of participants confirmed that the primary challenge they encounter, at varying rates, is the IFD’s inability to effectively communicate with PWD, including those who use sign language and Braille. Furthermore, they perceive a lack of proficiency and a failure to allocate sufficient time during sessions for patients with PWD. In addition, the participants held the belief that the healthcare program failed to provide adequate services and did not effectively implement the rights and legislation for PWD. Clearly, respondents agreed that one of the most important obstacles affecting doctors’ treatment of PWD is the lack of attendance at special education training workshops. The participants attributed this result to the absence of curricula in medical colleges that teach and educate doctors about the characteristics of special education. This leads to difficulties for doctors when dealing with PWD due to the inability to communicate effectively between them and how to control the behavior of PWD.

This finding aligns with the findings of Shabby et al. (8) and Woodman et al. (33) have highlighted the challenges faced by healthcare professionals in communicating with PWD. They found that doctors often lack the necessary skills to effectively interact with PWD, leading to feelings of discontent or marginalization, also, that doctors struggle to communicate with PWD due to their lack of qualification in dealing with them (9, 44). Additionally, Keller (7) demonstrated that the difficulties faced by IFD when communicating with PWD, citing their struggles with non-verbal communication, cognitive functions, and auditory or visual processing. To address these issues, interpreters, written materials, or assistive technology can be provided to improve the quality of life for PWD (27).

The majority of study results align with the current study’s findings, KSA’s medical schools and IFDs lack comprehensive disability curriculum and holistic approach to PWD care, highlighting the need for more training on the unique requirements of PWD in order to provide effective care. Research suggests that heightened exposure to disability issues and specialized training may improve these attitudes, as demonstrated by educational treatments (33, 45, 49). Furthermore, medical school curricula often lack of training programs to IFD to increase performance efficiency in dealing with PWD, despite progress in medical education reform. Special education and disability-specific curricula are still limited, necessitating a more comprehensive approach for future doctors (31, 48, 50). Iezzoni et al. (31) also recommended that as a solution to the problem of IFD bias against PWD, all levels of medical education must include more training on disability, including cultural competency for special education and etiquette. Also, provide private home care, standards of care in crises, and equitable care. At the same time, studies by (9), Keller (7), and Lee et al. (12) confirmed that PWD’s needs are not adequately addressed in the health sector due to a lack of educational workshops and training for doctors, affecting their ability to treat them effectively. Additionally, IFD faces financial resource shortages and time constraints in treating PWD, and Lee, highlight the need for additional coordination efforts and assistive technology acquisition to improve care in busy practices.

This study explores the opinions of participants on developing solutions to prepare medical workers to serve PWD. Participants also emphasize the importance of special education diplomas and curricula for instructing IFD and health sector professionals in dealing with PWD.

This is what the studies referred to Rao (74) and Al-Muntashri et al. (75) that special education programs can enhance medical curricula by incorporating disability studies, hands-on training, simulation exercises, fostering interdisciplinary collaboration, and providing training on technology and assistive devices. These strategies can lead to more inclusive practices for PWD, enhancing their understanding and treatment. Examples: Georgetown University Medical School in the USA, University of Birmingham in the UK, the University of California, San Francisco’s, King’s College London’s, University of Sydney’s, Harvard Medical School’s and Monash University in Australia offer specialized training on disability competency in healthcare, patient-centered care, and real-world case studies to integrate disability and inclusion training (16, 76). Furthermore, the respondents believe that having a special education specialist in every clinic can help doctors tackle challenges. Implementing this system is a promising idea, but global implementation is challenging due to resource constraints and training requirements. Also, there is a lack of resources that hinders access to specialized care for people with PWD, as APD (26) and GOV.SA (25) have shown that there is a shortage of specialized PWD rehabilitation clinics, limiting access to these facilities for those living in more isolated areas. Moreover, most participants agree that doctors should attend periodic training workshops, and some suggest motivating them to excel in handling special cases by offering material and moral incentives like an excellence allowance. They also recommend requiring doctors to complete sufficient courses and earn accredited certificates in this field. As one solution, the researcher suggests that it is necessary to consider creating a requirement to pass a licensing or final exam before completing their training and graduating from the IFDs. These exams should incorporate questions related to PWD knowledge and management to better prepare them for practice.

Most studies have agreed on the need to create training courses on how to deal with PWD that raises awareness among doctors, enhancing their ability to handle their unique needs. It incorporates disability etiquette, patient-centered learning, and interprofessional learning sessions. Post-curriculum training improves attitudes towards PWD, equipping them to handle their unique needs. This curriculum fulfills essential competencies, offers interprofessional learning opportunities, and integrates PWD into the educational framework (29). This finding is in agreement with that of Keller (7) and Lee et al. (12), that enhancing healthcare accessibility for PWD necessitates the advancement of healthcare professional training, increased access to assistive technologies, and the implementation of structural reforms. Furthermore, the US Department of Education (36) demonstrates that the curriculum enhanced the attitudes of IFD toward PWD following their post-curriculum training. Future IFD will therefore be prepared to address the specific needs of PWD.

## Conclusion

This study aimed to describe the extent to which IFD can effectively deal with PWD by understanding the trends and challenges that IFD face when working with PWD. The findings indicate that IFD require further training in the special education curriculum to effectively manage PWD. The majority of the study’s findings validated the information present in the existing literature. Identifying ways to improve doctors’ ability to provide health services for PWD was also a unique and perhaps underexplored point. We are creating a new curriculum to educate doctors on the psychological, linguistic, communication, and health aspects of dealing with PWD. Despite conducting this study across all IFD in Riyadh’s medical colleges, it’s crucial to concentrate on multiple specialties within these institutions. The questionnaire method may have been a limitation of the study. Future studies could explore this phenomenon further, using either a comparative or quasi-experimental approach to better understand and measure the ability of doctors to deal with PWD and improve their quality of health life.

## Limitations of the study

The study’s drawing of participants from a single geographical concentration posed a weakness that could potentially impact the reproducibility of the findings or generalizability of the study. Where the study is concentrated in Riyadh, which limits the generalizability of the findings to other regions within KSA, such as rural areas where healthcare challenges for PWD may differ. Another limitation of the study was the lack of Arab research on the ability of IFD to manage PWD in hospital settings. Multiple search engines were searched, but the research on the subject was very little, perhaps due to specialists’ lack of interest in linking the medical aspect with the academic aspect of special education. Furthermore, the delay and non-response of some participants in the sample to answer the questionnaire were limitations that caused delays for the researcher in collecting data, despite their constant reminders via email.

One of the most significant limitations of the study is its reliance on a single data collection tool, the questionnaire. This is because closed questions, which rely on the accuracy of participants’ responses, cannot provide accurate and valid answers. In contrast, interviews and field observations offer a comprehensive depiction of a phenomenon. To address these limitations, we posed an open question to the participants, enabling them to express themselves more deeply. The study’s limitation is its reliance on self-reported data, which may introduce biases like recall or social desirability bias. To mitigate these, measures like confidentiality and privacy were implemented, and questions were designed to be clear and avoid misunderstanding. However, the potential for bias remains, and future studies should use objective measurement methods or secondary data to validate the results.

## Implications for future research

Finally, future research should explore ways to enhance the education of health personnel, equipping them with the necessary skills to effectively manage PWD. This would be particularly beneficial and effective in fostering an inclusive, healthy environment for PWD in hospitals. Furthermore, we should concentrate on conducting additional research to develop contemporary programs and curricula in special education, which will equip health staff with the necessary skills. Future collaboration between health and special education specialists is crucial for achieving integration. Future studies should also concentrate on understanding the capacity of doctors from various specialties to manage PWD.

## Data Availability

The data analyzed in this study is subject to the following licenses/restrictions: maintaining confidentiality of participants’ data by coding questionnaires. Requests to access these datasets should be directed to aa_safar@ksu.edu.sa.
