# Supplementary material for: The extent to which family doctors have the ability to deal with patients with disabilities
Source: Front Med (Lausanne). 2025 Apr 16;12:1524123. doi: 10.3389/fmed.2025.1524123 (PMC12040965; doi:10.3389/fmed.2025.1524123)
Supplement: Supplementary file 1 [file Data_Sheet_1.pdf]

Appendix 1

Appendix (1) The part quoted from the scale (Chadd & Pangilinan, 2011 as stated in Bu & et al., 2016)

TABLE 2 Item scores on the disability attitudes in healthcare survey[19] for intervention and control groups

	Control	Intervention	*P*[a]
1. People with disabilities are pleasant to be with.	3.51	3.87	**0.001**
2. If I had the choice, I would rather see able-bodied patients than patients with disabilities.[b]	2.96	3.13	**0.05**
3. It is society's responsibility to provide care for its persons with disabilities.	4.13	4.17	0.60
4. Patients with disability improve with treatment.	3.89	3.92	0.63
5. Medical care for PWDs uses up too many resources.[b]	3.67	3.77	0.25
6. Taking a medical history from a patient with disability is frequently an ordeal.[b]	3.17	3.37	**0.02**
7. People with disability, in general, do not contribute much to society.[b]	4.00	4.28	**0.001**
8. People with disability don't contribute their fair share toward paying for their health care.[b]	3.67	3.83	0.07
9. I will welcome patients with disability into my practice.	4.22	4.35	**0.04**
10. If handled properly, the patient with disability can be seen as quickly as any other patient.	3.43	3.47	0.62
11. Understanding my patients with disability is valuable to me as a physician.	4.30	4.38	0.14
12. Patients with disability are better off in nursing homes.[b]	3.90	4.11	**0.005**
13. Medicare offers adequate compensation for care of patients with disability.	2.59	2.67	0.27
14. The treatment of patients with disability is too time consuming.[b]	3.73	3.85	0.10
15. More training is needed to prepare health practitioners to provide care to the patient with disability.	3.80	3.90	0.17
16. It is unglamorous to care for people with disability.[b]	3.76	3.89	0.12
17. Treatment of people with disability is hopeless.[b]	4.38	4.42	0.52

The 5-point Likert scale was scored with weights from strongly disagree = 1 to strongly agree = 5.

[a]Independent *t* test.

[b]The scores for negatively worded items were inverted so that higher scores indicate stronger disagreement toward these statements.

Boldface indicates statistical significance.

Appendix (2) The scale after modification

N	Item	Percent %			Average	Std. Deviation	Frequency
		Yes	Some time	No			
1	I am happy to be with PWD.	51	36	11	2.4	0.72	3
2	If I had the choice, I would prefer to work with healthy patients instead of PWD	40	32	26	2.1	0.83	2
3	The community has the responsibility to provide care for PWD.	81	11	0	2.7	0.61	3
4	PWD improve with treatment	59	38	0	2.6	0.59	3
5	Medical care for PWD consumes a lot of resources	50	28	21	2.1	0.72	2
6	Obtaining a medical history from a PWD is often a disaster and very difficult.	13	52	34	1.8	0.67	2
7	PWD generally do not contribute much to society.	0	35	59	1.5	0.61	1
8	The healthcare costs for PWD are expensive compared to an average person, and this is unfair.	35	21	43	1.9	0.90	2
9	I will welcome PWD in my clinic.	92	7	1	2.9	0.41	3
10	If the PWD is handled properly, they can be seen quickly like any other patient.	67	26	7	2.6	0.63	3
11	The extent to which my PWD understand is valuable to me as a doctor.	96	4	0	2.9	0.31	3
12	PWD are better off in specialized care facilities.	28	43	27	2.9	0.85	2
13	The healthcare program provides appropriate compensation for the care of PWD	31	52	15	2.1	0.69	2
14	Treating PWD takes an extremely long time.	22	60	17	2.0	0.65	2

**15**	There is a need for more training to prepare healthcare practitioners to provide care for PWD	86	13	0	2.9	0.41	3
**16**	Caring for PWD is not enjoyable.	11	28	59	1.5	0.71	1
**17**	Treatment for PWD is hopeless.	0	11	82	1.1	0.34	1
	**Total axis score**			152	respondents		
